# Mechanistic Phenotypes: An Aggregative Phenotyping Strategy to Identify Disease Mechanisms Using GWAS Data

**DOI:** 10.1371/journal.pone.0081503

**Published:** 2013-12-12

**Authors:** Jonathan D. Mosley, Sara L. Van Driest, Emma K. Larkin, Peter E. Weeke, John S. Witte, Quinn S. Wells, Jason H. Karnes, Yan Guo, Lisa Bastarache, Lana M. Olson, Catherine A. McCarty, Jennifer A. Pacheco, Gail P. Jarvik, David S. Carrell, Eric B. Larson, David R. Crosslin, Iftikhar J. Kullo, Gerard Tromp, Helena Kuivaniemi, David J. Carey, Marylyn D. Ritchie, Josh C. Denny, Dan M. Roden

**Affiliations:** 1 Department of Medicine, Vanderbilt University, Nashville, Tennessee, United States of America; 2 Department of Pediatrics, Vanderbilt University, Nashville, Tennessee, United States of America; 3 Department of Epidemiology and Biostatistics, University of California San Francisco, San Francisco, California, United States of America; 4 Biomedical Informatics, Vanderbilt University, Nashville, Tennessee, United States of America; 5 Department of Cancer Biology, Vanderbilt University, Nashville, Tennessee, United States of America; 6 Center for Human Genetics Research, Vanderbilt University, Nashville, Tennessee, United States of America; 7 Essentia Institute of Rural Health, Duluth, Minnesota, United States of America; 8 Center for Genetic Medicine, Northwestern University Feinberg School of Medicine, Chicago, Illinois, United States of America; 9 Departments of Medicine (Medical Genetics) and Genome Sciences, University of Washington, Seattle, Washington, United States of America; 10 Group Health Research Institute, Seattle, Washington, United States of America; 11 Division of Cardiovascular Diseases, Mayo Clinic, Rochester, Minnesota, United States of America; 12 The Sigfried and Janet Weis Center for Research, Geisinger Health System, Danville, Pennsylvania, United States of America; 13 Department of Biochemistry and Molecular Biology, Penn State University, University Park, Pennsylvania, United States of America; University of Pennsylvania, United States of America

## Abstract

A single mutation can alter cellular and global homeostatic mechanisms and give rise to multiple clinical diseases. We hypothesized that these disease mechanisms could be identified using low minor allele frequency (MAF<0.1) non-synonymous SNPs (nsSNPs) associated with “mechanistic phenotypes”, comprised of collections of related diagnoses. We studied two mechanistic phenotypes: (1) thrombosis, evaluated in a population of 1,655 African Americans; and (2) four groupings of cancer diagnoses, evaluated in 3,009 white European Americans. We tested associations between nsSNPs represented on GWAS platforms and mechanistic phenotypes ascertained from electronic medical records (EMRs), and sought enrichment in functional ontologies across the top-ranked associations. We used a two-step analytic approach whereby nsSNPs were first sorted by the strength of their association with a phenotype. We tested associations using two reverse genetic models and standard additive and recessive models. In the second step, we employed a hypothesis-free ontological enrichment analysis using the sorted nsSNPs to identify functional mechanisms underlying the diagnoses comprising the mechanistic phenotypes. The thrombosis phenotype was solely associated with ontologies related to blood coagulation (Fisher's p = 0.0001, FDR p = 0.03), driven by the *F5, P2RY12* and *F2RL2* genes. For the cancer phenotypes, the reverse genetics models were enriched in DNA repair functions (p = 2×10−5, FDR p = 0.03) (*POLG/FANCI, SLX4/FANCP, XRCC1, BRCA1, FANCA, CHD1L*) while the additive model showed enrichment related to chromatid segregation (p = 4×10−6, FDR p = 0.005) (*KIF25, PINX1*). We were able to replicate nsSNP associations for *POLG/FANCI, BRCA1, FANCA* and *CHD1L* in independent data sets. Mechanism-oriented phenotyping using collections of EMR-derived diagnoses can elucidate fundamental disease mechanisms.

## Introduction

A single mutation can alter cellular and global homeostatic mechanisms and give rise to multiple clinical diseases, as seen in coagulopathies and cancer syndromes [1]–[3]. This genetic pleiotropy is not captured by current forward genetic approaches, such as genome wide association studies (GWAS) for specific diseases, which typically narrow case definitions to reduce genetic heterogeneity and improve the signal-to-noise ratio [4]. By evaluating only a small portion of a variant's phenotypic spectrum, these approaches may underestimate true genetic effect sizes. Quantitative approaches to address genetic pleiotropy typically utilize either data reduction methods [5]–[7] or post-hoc evaluation of association statistics derived from individual phenotypes [8]. These approaches, however, either require correlated traits or are limited to a relatively few traits [9]. Hence, they are not well-suited for complex phenotypes such as cancer where tumor multiplicity within individuals is rare and the phenotypic spectrum is broad. An alternative analytic approach is a reverse genetics model, which characterizes the phenotypic consequences of a known genetic mutation [10]. However, current implementations such as Phenome-Wide Association Study (PheWAS), which serially tests for associations between common polymorphisms and hundreds of clinical disease entities [11], also rely on discrete, pre-specified phenotypes.

To leverage genetic pleiotropy for single nucleotide polymorphism (SNP) discovery, we propose an analytical approach employing “mechanistic phenotypes”. We define a mechanistic phenotype as the collection of all clinical diseases that arise from the perturbation of a discrete cellular mechanism. Unlike current approaches to modeling genetic pleiotropy, all constituent diseases comprising a mechanistic phenotype are considered equivalent, even though they may be clinically disparate. By assuming a common underlying cellular mechanism among the diseases, a mechanistic phenotype incorporates a broad disease spectrum into a single phenotype definition, which may increase the power to detect a true functional association with any underlying genetic variants, if the variant interrupts a cellular mechanism common to the phenotypes. A test of this idea requires datasets that encompass many potentially mechanistically-related diagnoses, an attribute characteristic of electronic medical record (EMR) systems [11].

We hypothesized that SNPs truly associated with mechanistic phenotypes disrupt basic cellular or physiological mechanisms and that these mechanisms would be delineated by identifying functional commonalities among genes containing SNPs with the strongest associations. We tested this hypothesis with two distinct disease processes, thrombosis and tumorigenesis, and defined mechanistic phenotypes comprised of collections of thrombosis or cancer-related diagnoses. We identified associations between low minor allele frequency (MAF) non-synonymous SNP (nsSNP) variants and mechanistic phenotypes derived from diagnoses extracted from EMR data. We analyzed low frequency nsSNP variants since they may contribute substantially to genetic heritability and can have large effect sizes, thereby enhancing the effectiveness of our approach [12]–[14]. We employed a two-step discovery approach. In the first step, nsSNPs were first sorted by the strength of their association with the mechanistic phenotypes, as measured by association p-values. In the second step, the most strongly associated nsSNPs were tested for enrichment in cellular ontologies. We compared four association approaches: standard recessive and additive forward genetics models and two reverse genetics models. The reverse genetics models perform association testing using subsets of constituent diseases that meet pre-defined selection criteria. These models analyzed only homozygotes, as we hypothesized that the effects of a nsSNP would be strongest among minor allele homozygotes, and this effect would be best detected when contrasted with common allele homozygotes. We report that a hypothesis-free ontological enrichment discovery approach showed that hemostatic mechanisms were specifically associated with the thrombosis phenotype and mechanisms underlying genomic stability (DNA repair and chromatid segregation) were exclusively associated with cancer. These results demonstrate that mechanistic phenotypes using low frequency nsSNPs can identify underlying disease processes and functionally enriched sets of candidate genes.

## Materials and Methods

### Ethics Statement

The genetic materials at Vanderbilt were accrued through leftover blood collected through routine clinical testing. The Vanderbilt BioVU resource operates as nonhuman subjects research according to the provisions of *Code of Federal Regulations 45*, part 46, as described previously [15]. Individuals at other eMERGE sites were consented as part of the DNA biobank at each site [16]. This study was approved by the Institutional Review Board at each site (Group Health Cooperative, University of Washington, Marshfield Clinic, Mayo Clinic, Northwestern University, Vanderbilt University and Geisinger Health System).

### Study population

3,009 white and 1,655 African-American (AA) subjects who had previously been genotyped at Vanderbilt University Medical Center (VUMC) were used in the thrombosis and cancer analyses, respectively. Subjects were drawn from VUMC's BioVU resource, a de-identified collection of patients whose DNA was extracted from discarded blood and linked to phenotypes through a de-identified electronic medical record [15]. The subjects belonged to two curated cohorts with pre-existing GWAS data: the Vanderbilt Genome Electronic medical Records (VGER) project (n = 1,217) within the electronic Medical Records and Genomics (eMERGE) network [16]; and an ongoing study (Vanderbilt Electronic Systems for Pharmacogenomic Assessment; VESPA) examining the genomics of drug response phenotypes (n = 3,447). Race assignment was determined using STRUCTURE [17], with whites and AAs defined as a >90% probability of being in the CEU or YRI clusters, respectively, using a HapMap population as the reference.

### SNP Selection

Genotype data were acquired on the Illumina 1M-Duo (n = 433 nsSNPs), Infinium Exome BeadChip (n = 83 nsSNPs) and Omni1_QUAD (n = 350 nsSNPs) platforms for AA subjects; and the Omni1_QUAD platform for white subjects. Each dataset was separately cleaned. Quality control was performed on the Exome BeadChip data by VANGARD (Vanderbilt Technologies for Advanced Genomics Analysis and Research Design) using a 2 stage process involving Genome studio and PLINK. The samples used in this study were analyzed in conjunction with over 20,000 other BeadChip samples. In Genome Studio, SNPs were clustered and to ensure correctness, manual reclustering was performed based on quality control measurements such as GenTrain Score, Cluster Separation, Call Freq scores. Samples were then evaluated for heterozygous consistency rate between duplicated samples, heterozygous consistency rate between HAPMAP samples, their 1000 Genome genotyping calls and genotype consistency between duplicated SNPs on the SNP chip. All samples were evaluated for gender mismatches, SNPs failing concordance with HapMap, Mendelian errors, duplicate identification and exclusion of subjects more closely related than half-siblings. SNPs and samples with a call rate <98% were excluded. nsSNPs with a MAF less than 10% in both white and African-American races and with >9 (thrombosis in AAs) or >10 (cancer in whites) homozygotes for the minor allele were selected. This MAF threshold was selected in order to select nsSNPs with relatively low frequencies and with sufficient minor allele homozygotes for analyses based on the number of samples available for analysis. For whites, 833 nsSNPs in 748 distinct genes where identified. For AAs, 433 nsSNPs (404 genes) met the selection criteria. Six and seven nsSNPs in whites and AAs, respectively, had a Hardy-Weinberg p<0.001. The mean MAF in whites was 7.6% (range 3.7–10%) and 8.5% (range 5.2 to 9.9%) in AAs. A description of the nsSNPs analyzed and exclusions is shown in Tables S1, S2, S3 in File S1.

### Phenotype creation

Clinical diagnoses and problems were extracted from the VUMC Synthetic Derivative, a de-identified image of the Vanderbilt EMR [15]. Diagnoses were derived from ICD-9 codes and physician-maintained problem lists. Problem lists were manually reviewed and diagnoses were mapped to the most closely matching ICD-9 code. A total of 673 clinical non-benign cancer codes and 117 codes related to blood vessel-occlusive disease (thrombosis) were identified. These codes were aggregated into 191 and 21 groups of related cancer and thrombosis codes, respectively (see Tables S4 and S5 in File S1 for groupings).

A conceptual model of mechanistic phenotypes is shown in Figure S1. A thrombosis mechanistic phenotype was defined using all 21 thrombosis groupings. Five cancer mechanistic phenotypes were defined by assigning the 191 aggregated coding groups to one or more mechanistic phenotype groupings based on common clinical or epidemiological features: [ALL] – all cancer codes, [HEM] – all codes for hematological malignancies (rationale: cancers with a common cellular origin); [CA] – all diagnosis codes for primary, non-hematological malignancies, excluding skin cancers (rationale: tumors that require specific mechanisms such as angiogenesis); [SKN] –codes related to melanoma, basal cell carcinoma and squamous cell carcinoma (rationale: tumors that arise due to unique exposures such as UV radiation); [MET] – all codes for metastatic/secondary cancers (rationale: tumors with mechanisms critical for metastasis such extravasation).

### SNP association models

**Figure 1:**
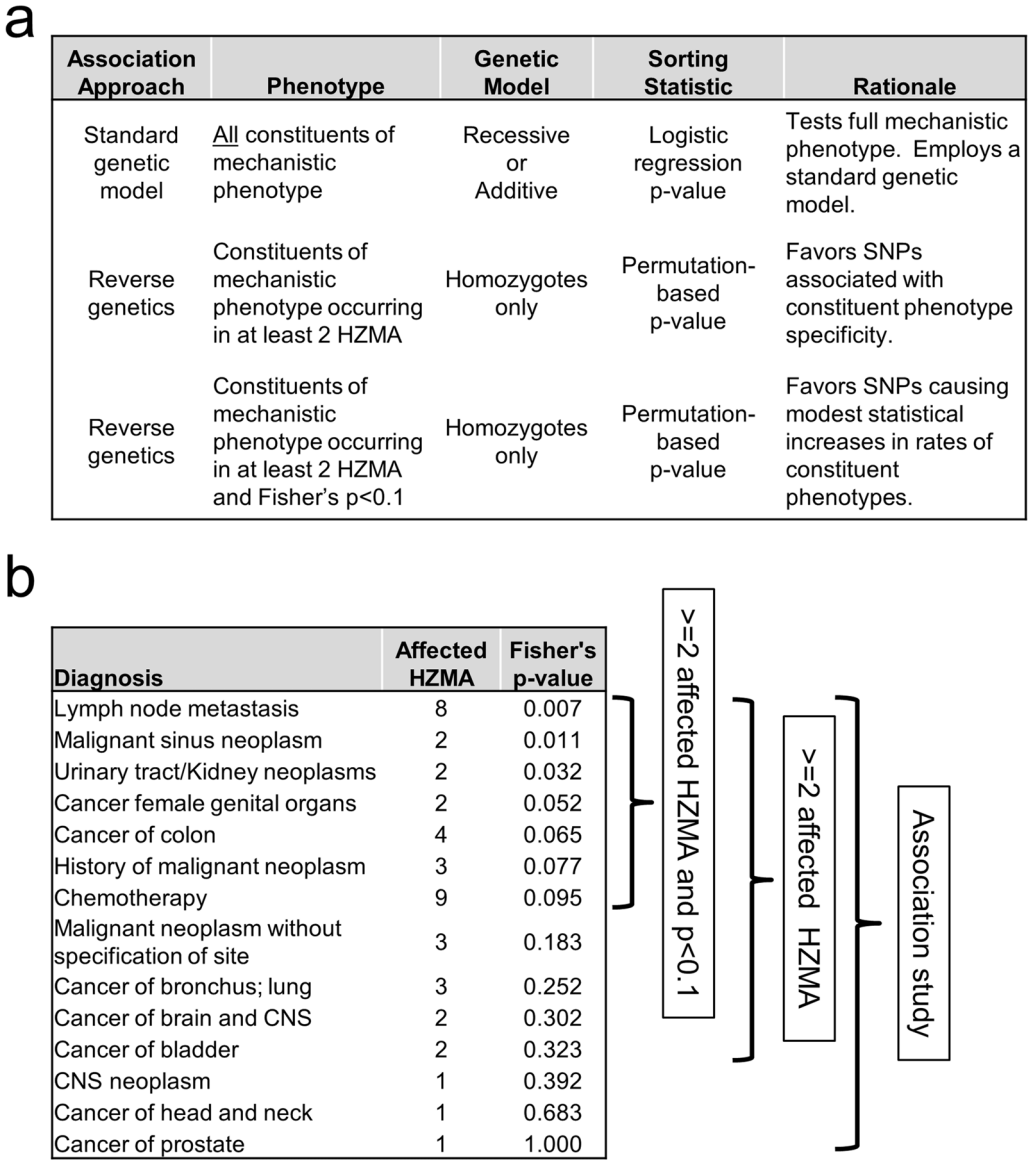
Overview of the nsSNP association approaches. Panel (a) describes key features of the SNP association approaches used. Panel (b) shows, for a single hypothetical SNP, how assignment of affection status for homozygotes for the minor allele (HZMAs) varies by the approaches. The table lists cancer codes present among the HZMAs, the number of HZMAs that have the cancer code and the Fisher's p-value comparing the proportion of affected HZMAs with the cancer to the proportion in the common allele homozygotes. For this example, all of the listed cancers are assumed to be constituents of the mechanistic phenotype. For the standard genetic models, all subjects with any of the cancers are classified as cases. In contrast, the 2 reverse genetics approaches only analyze subsets of these subjects with cancers meeting pre-specified criteria, as designated by the brackets.

Four analytical approaches (standard recessive or additive genetic models; and 2 reverse genetics approaches) were used to quantify associations between SNPs and the mechanistic phenotypes (Figure 1).

#### Additive and recessive models

For each mechanistic phenotype, cases were those subjects having a diagnosis that was a constituent of the mechanistic phenotype (Tables S4 and S5 in File S1). Controls were those without a constituent diagnosis. For each SNP and mechanistic phenotype, association p-values were generated using multivariable logistic regression employing an additive or recessive genetic model and adjusting for gender, age and dataset from which the subjects were derived. None of the top five principal components (PC) from each population was associated with a mechanistic phenotype and PCs were not included in the statistical models. QQ plots for the additive models testing the associations between all SNPs on the genotyping platforms and the mechanistic phenotypes did not show a pattern of genome-wide inflation (see Figure S2 and Figure S3). Only SNPs with an odds-ratio>1 were used in enrichment analyses, as the goal of these analyses was to identify low-frequency variants associated with an increased risk of the phenotype.

#### Reverse genetics models

In general, these approaches differ from the recessive genetic model in that only a subset of the constituent diagnoses for a mechanistic phenotype are used to compute association p-values. In addition, only homozygotes for the common and minor alleles were analyzed, as nsSNPs with an additive mode of action would be expected to have higher rates of affected heterozygotes, which would attenuate the association between the nsSNP and the phenotype for these models.

Approach 1 (>1 affected): This approach computed association p-values for a SNP based only on constituent diagnoses of the mechanistic phenotype that occurred more than once among the homozygotes for the minor allele. For instance, if 10 diagnoses were present among the homozygotes, but only 5 occurred in more than 1 subject, only those 5 diagnoses were evaluated. For each SNP, the number of homozygotes that have any of the multiply-occurring diagnosis was tallied. A p-value representing the likelihood of observing a tally equal or greater than this tally was computed using a permutation-based approach. Specifically, for each SNP, the genotypes were permuted (i.e. the genotypes were randomly reassigned among the subjects) 10,000 times. Permuting genotypes has the net effect of preserving the overall numbers of subjects (and MAF) for a given SNP genotype. Permutations were stratified by study, age and gender. For each permutation, a new tally was computed for the newly-assigned minor allele homozygotes based on the diagnoses that occurred more than once among these subjects. The association p-value is the proportion of the permutations with tallies equaling or exceeding the original data. A p-value was computed for each mechanistic phenotype and SNP.

Approach 2 (>1 affected and Fisher's p<0.1): This approach was identical to approach 1, with the exception only multiply-occurring constituent diagnoses that had a univariate Fisher's Exact p-value<0.1 (testing the association of the diagnosis between the minor allele and common allele homozygotes) were used to compute the tallies. The Fisher's p-values were not adjusted for multiple testing as their function was to ascertain whether a given diagnosis was represented among minor allele homozygotes at rates higher than would be expected by chance, based on the frequency among the common allele homozygotes. The relatively-high p-value threshold of 0.1 was selected to exclude those constituent diagnoses that were present among minor allele homozygotes at levels generally expected by chance. Based on analyses of permuted cancer data sets, this threshold excluded ∼67% of all constituent diagnoses.

### Simulation studies

Simulation studies were conducted to estimate the expected number of false positives (type I error) associated with the reverse genetics approaches. Randomized sets of SNPs with the same MAF distribution as the SNPs in the thrombosis data set were generated by permuting all genes in that data set 1,000 times, giving n = 433,000 randomized SNP genotypes. Similarly, SNP genotypes were permuted 500 times in the cancer data set, giving 416,500 randomized SNPs. The association p-value for each permuted SNP and the thrombosis or all cancer [ALL] mechanistic phenotypes, respectively, was computed using the two reverse genetics methods. Type 1 error rates for cut-offs between 0.1 and 0.0001 are the proportion of permuted SNPs with association p-values falling below these cut-offs.

To characterize how the reverse genetics models would perform in the presence of a disease-causing SNP, 10,000 random samples of 13 phenotyped subjects were drawn from the thrombosis data set. The number of subjects selected was the median number of minor allele homozygotes for the 433 nsSNPs analyzed in this data set. Four scenarios representing possible ways that a SNP might cause additional cases were simulated. For each scenario, 5 of the 13 subjects were assigned to be affected with a constituent diagnosis from the thrombosis mechanistic phenotype (Table S4 in File S1). While these 5 subjects did not have the newly assigned diagnosis, they may have had another constituent diagnosis. New diagnosis assignment was made for four scenarios: 1) each of the 5 subjects was assigned a constituent diagnosis randomly selected from the list of diagnoses; 2) each of the 5 subjects was assigned a diagnosis selected with probability equal to the frequency of the diagnosis in the data set; 3) each of the 5 subjects was assigned a diagnosis already present among the 13 subjects; and 4) each of the 5 subjects was assigned the same diagnosis, selected randomly from the list of diagnoses. Under the first 3 scenarios, it is possible that each of the 5 subjects was assigned a different diagnosis. P-values for each reverse genetics model were computed, as described above. Receiver-operator curve (ROC) and area under the curve (AUC) analyses were used to compare the relative sensitivity and specificity of the reverse genetics models in distinguishing SNPs with additional cases to SNPs without additional cases. As a reference, a model which computed p-value based on counts all affected subjects was also tested. This model was similar to a recessive genetic model, with the exception that p-values were estimated by permutation.

### Ontological enrichment

To test the primary hypothesis that nsSNPs most strongly associated with the mechanistic phenotype (i.e. nsSNPs with the lowest p-values) have common functionalities, the top-ranked SNPs were evaluated for ontological enrichment. All Biological Process (BP) ontologies for each gene within each data set were downloaded using the DAVID software program [18] and are found in Tables S6 and S7 in File S1 (n = 1856 and n = 2623 distinct ontologies for the thrombosis and cancer sets, respectively). Enrichment p-values were computed by sequentially selecting and testing incremental numbers of the top-ranked genes from a list of genes sorted by the association p-values between the nsSNP in the gene and the mechanistic phenotype. All genes containing nsSNPs with an association p<0.05 were sequentially tested. In addition, the top 50 genes in the top nsSNPs from GWAS analyses of thrombosis genes in AAs and the all cancer [ALL] phenotype in whites using all SNPs were also tested for enrichment. A Fisher's exact test was used to test for ontological enrichment between the top-ranked selected genes and all other genes. For each ontology, the lowest Fisher's p-value from the sequential tests was identified. A low Fisher's p-value would indicate that functionally related genes were relatively more strongly associated with the phenotype. These p-values were adjusted for multiple testing using a Benjamini-Hochberg (B–H) FDR correction based on all ontologies present among the genes. Only those ontologies with a B–H FDR p<0.05 and with more than 1 associated gene were considered significantly associated. For comparisons between models, some results tables show enrichment results for ontologies that did not meet the criteria for significant enrichment.

### Testing cancer associations in external datasets

SNPs associated with DNA repair ontologies were evaluated in 2 independent data sets. The first contained 3,928 additional self-reported white subjects genotyped on the Illumina Omni1_QUAD as part of the VESPA (Vanderbilt Electronic Systems for Pharmacogenomic Assessment) study. The second data set came from five sites participating in the eMERGE-I&II consortium (Marshfield Clinic, Northwestern University, Mayo Clinic, Group Health Research Institute, Geisinger Health System) and contained 14,049 self-reported white subjects who underwent genotyping using the Illumina Human660W-Quadv1_A or HumanOmniExpress-12v1.0 platforms [19]. Age, gender, self-reported race, decade of most recent diagnosis and ICD-9 codes were available for each subject. For the recessive model, each homozygous minor allele carrier, 20 controls homozygous for the common allele were selected, matched on age of diagnosis, gender and eMERGE site. Only those SNPs that had > = 10 minor allele homozygotes were analyzed. Exact logistic regression was used to test the associations between each SNP and phenotype. For the additive model, all subjects were analyzed. A p<0.05 was considered statistically significant.

### Data analysis

All quality control analyses were performed using PLINK v1.07 [20]. P-values for the additive and recessive genetic models were also computed using PLINK. All other analyses were performed using SAS v9.3 (SAS Institute, Cary, NC). Biological Process (BP) ontologies were downloaded using the Database for Annotation, Visualization and Integrated Discovery (DAVID) v6.7 tool set [18].

## Results

### Simulation Studies

We tested two reverse genetics models that computed association p-values for a mechanistic phenotype based on features of the constituent diagnoses comprising the phenotype (see Methods and Figure 1). Phenotype data for the simulation studies was derived from a set of 1,655 AA subjects for the thrombosis mechanistic phenotype and from 3,009 whites for the all cancer [ALL] mechanistic phenotype (Table 1). Type I error rates were computed to ascertain the expected distribution of p-values from the models under the null hypothesis. In general, empirical type 1 error rates approximated the association p-values (for instance, the type 1 error rate for a SNP with a p<0.001 was 0.0011 and 0.0016 in the thrombosis data set for the two models, respectively [Table S8 in File S1].

**Table 1:** Population characteristics.

	Thrombosis study	Cancer study
Total subjects (n)	1655	3009
No. males (%)	596 (36.0)	1699 (56.5)
No. females (%)	1059 (64.0)	1310 (43.5)
Mean (SD) age	52.3 (17.7)	51.4 (18.7)
Thrombosis diagnoses: n (%)		
All thrombosis phenotypes	454 (27.4)	
Long-term anticoagulation	179 (10.8)	
Stroke	165 (10.0)	
Acute myocardial infarction	149 (9.0)	
Venous thrombosis	116 (7.0)	
Thrombotic pulmonary disease	47 (2.8)	
Other disorders^1^	44 (2.7)	
Arterial thrombosis	36 (2.2)	
Spontaneous abortion	31 (1.9)	
Cancer diagnoses: n (%)		
All cancers		1276 (42.4)
Non-hematological, primary (CA)		1076 (35.8)
Secondary/metastases (MET)		362 (12.0)
Hematological (HEM)		371 (12.3)
Skin (SKN)		109 (3.6)

= 16), Primary hypercoagulable state (n = 13), Budd-Chiari syndrome (n = 3), Thrombophlebitis migrans (n = 1), other congenital deficiencies (n = 2), congenital factor IX disorder (n = 1), other coagulation defects (n = 6), congenital factor VIII disorder (n = 3). (1) Includes: Defibrination syndrome (n

**Figure 2:**
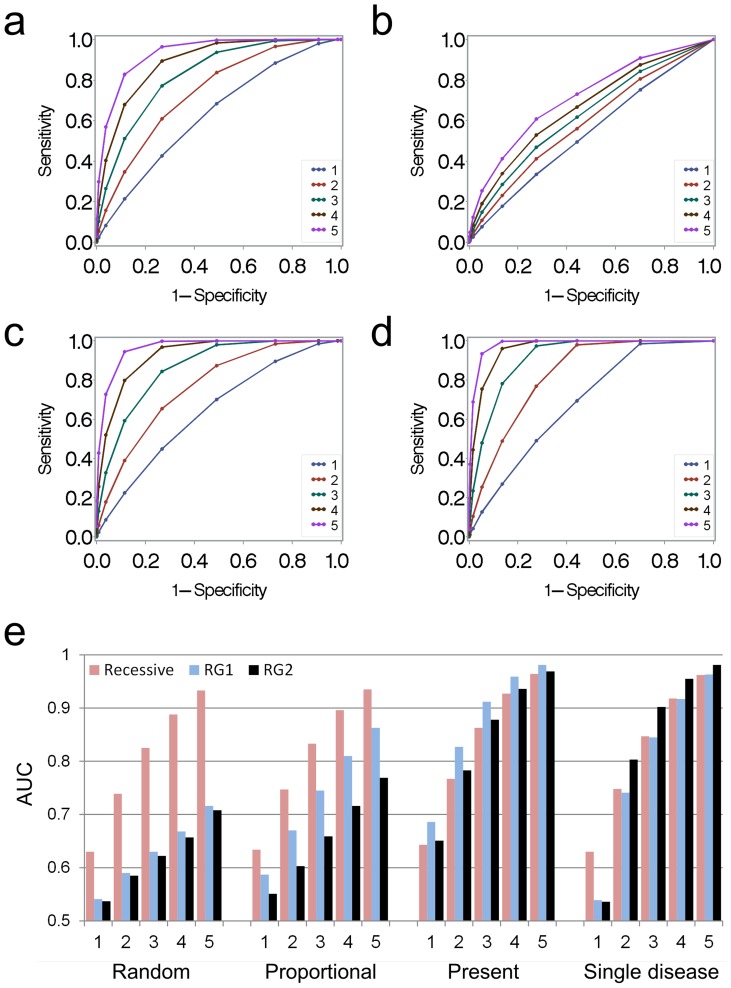
ROC analyses for simulation studies. Analyses are based on 10,000 random samples of 13 phenotyped subjects drawn from the thrombosis data set. ROC curves show sensitivity and specificities based on association p-values when one to five subjects were assigned to be affected with a constituent disease, as compared to association p-values associated with no additional cases. Panels (a) and (b) show ROC curves based on p-value associations for the recessive and reverse genetics models (with >2 affected subjects per constituent phenotype), respectively, when five subjects were assigned a random constituent disease. Each line corresponds to the number of additional subjects assigned a disease. Panels (c) and (d) represent the same models, respectively, for subjects assigned a disease already present among the 13 subjects in the random sample. Panel (e) summarizes AUC values from ROC curves for the recessive, reverse genetics with >2 affected subject (RG1) and reverse genetics with >2 affected and p<0.1 (RG2) models under the four simulations conditions tested. The number of the x-axis refers to the number of additional subjects assigned an affection status for each simulation scenario.

Simulation analyses were also used to characterize how the reverse genetics models would perform under different scenarios in which an SNP caused thrombotic disease. ROC analyses were used to compare the sensitivity and specificity of the models when one to five additional subjects among random samples of 13 phenotyped subjects were assigned to be affected with a thrombotic disease. A recessive genetic model performed significantly better than the reverse genetics models at discriminating SNPs that caused additional cases of a random constituent disease (AUC of 0.83 versus 0.63 and 0.62 respectively, with 3 additional cases,) (Figures 2a, b, 2e and Figure A in Figure S4). Similar results were observed when a SNP caused additional cases with a risk proportional to the frequency of the constituent disease in the dataset, though the reverse genetics models performed somewhat better (AUC of 0.83 versus 0.75 and 0.66, respectively) (Figure 2e and Figures A, B, C in Figure S5). In contrast, the reverse genetics models performed better when a SNP gave rise to multiple cases of constituent diseases (AUC of 0.86 versus 0.91 and 0.88, respectively) (Figure 2c, d, 2e and Figure B in Figure S4). In general, the reverse genetics model that computed association p-values using only those constituent diagnoses from the mechanistic phenotype that occurred more than once in the minor allele homozygotes performed better than model that computed p-values based on constituent diagnoses with a Fisher's p<0.1. The exception was for SNPs simulated to add additional cases that had a single, common diagnoses, where the latter model performed modestly better than all other models (0.85 versus 0.90, respectively) (Figure 2e and Figures D, E, F in Figure S5). In summary, these simulation studies demonstrate that the performance of the models varied based on the pattern of the constituent diseases comprising the mechanistic phenotype. The reverse genetic models performed best when the SNP demonstrated a degree of specificity for one or more constituent phenotypes. In contrast, these models performed poorly when a SNP caused additional cases of less frequently occurring constituent diseases.

### Identification of thrombosis ontologies in African Americans

We next tested these models in the AA thrombosis data set using 433 nsSNPs (404 genes) selected solely for MAF<0.1 and represented on common GWAS platforms. The distribution of p-values from association analyses between the nsSNPs and both reverse genetics models did not fall out of the range of expectation based on simulation studies of type 1 error rates (Table S8 and Tables S9 and S10 in File S1 for the association p-values for each nsSNP). Hence, candidate nsSNPs could not be identified on the basis of being statistical outliers. We next tested whether the nsSNPs most strongly associated the thrombosis phenotype had common functionalities based on ontological enrichment. While both models showed the strongest enrichment in ontologies related to blood coagulation, only the reverse genetics model that selected for >2 diagnoses demonstrated enrichment that beat FDR thresholds (Table 2, Tables S11 and S12 in File S1). The enriched ontologies were related to blood coagulation (Fisher's exact p = 0.0001), driven by coagulation factor *F5* [21], platelet receptor *P2RY12* [22] and the thrombin cofactor *F2RL2* [23]. We used the same approach using standard recessive and additive models. The recessive model showed enrichment in coagulation ontologies, but these did not beat FDR thresholds (Table 2, Tables S13 and S14 in File S1). In contrast, the additive model was not significantly enriched in any ontology (Tables S15 and S16 in File S1). A GWAS of all SNPs on the genotyping platforms using an additive model did not show any associations with genome-wide significance (Figure S2). When we took all missense nsSNPs from the full GWAS and looked for ontological enrichment among the top 50 genes, there were no significantly enriched ontologies (see Tables S17 and S18 in File S1 for the genes examined and enrichment analyses). The top ontologies, while not related to blood coagulation, were driven in the coagulation factor *F2/thrombin*.

**Table 2:** Enriched ontologies for genes associated with the thrombosis phenotype in AAs.

Model/GO code	GO Term	P-value threshold^1^	Genes below threshold^2^	Genes with ontology^3^	Fisher's exact p-value	FDR p-value	Genes
**Reverse Genetics Model (>2 affected subjects)**							
GO:0007596	blood coagulation	0.012	8	3	0.0001	0.03	*P2RY12, F5, F2RL2*
GO:0007599	hemostasis	0.012	8	3	0.0001	0.03	*P2RY12, F5, F2RL2*
GO:0042060	wound healing	0.012	8	3	0.0001	0.03	*P2RY12, F5, F2RL2*
GO:0050817	coagulation	0.012	8	3	0.0001	0.03	*P2RY12, F5, F2RL2*
GO:0050878	Regulation of body fluids	0.012	8	3	0.0001	0.03	*P2RY12, F5, F2RL2*
GO:0009611	wound response	0.03	14	4	0.001	0.4	*P2RY12, F5, F2RL2, PTAFR*
GO:0019932	2nd msgr signal	0.03	14	3	0.002	0.4	*P2RY12, GPRC6A, PTAFR*
GO:0007243	protein kinase cascade	0.011	7	2	0.005	0.57	*P2RY12, SH2D3A*
GO:0007242	intracellular signaling cascade	0.03	14	4	0.008	0.69	*P2RY12, SH2D3A, GPRC6A, PTAFR*
GO:0007166	cell surface receptor	0.03	14	5	0.009	0.82	*P2RY12, ADAM22, F2RL2, GPRC6A, PTAFR*
**Reverse Genetics Model (>2 affected subjects and Fisher p<0.1)**							
GO:0007596	blood coagulation	0.011	5	2	0.001	0.6	*P2RY12, F5*
GO:0007599	hemostasis	0.011	5	2	0.002	0.6	*P2RY12, F5*
GO:0042060	wound healing	0.011	5	2	0.002	0.6	*P2RY12, F5*
GO:0050817	coagulation	0.011	5	2	0.002	0.6	*P2RY12, F5*
GO:0050878	Regulation of body fluids	0.011	5	2	0.002	0.6	*P2RY12, F5*
GO:0003001	signal involved in cell-cell signaling	0.03	11	2	0.006	0.78	*LEP, UNC13B*
**Recessive Model**							
GO:0007596	blood coagulation	0.033	13	3	0.0005	0.16	*P2RY12, F5, F2RL2*
GO:0007599	hemostasis	0.033	13	3	0.0005	0.16	*P2RY12, F5, F2RL2*
GO:0042060	wound healing	0.033	13	3	0.0005	0.16	*P2RY12, F5, F2RL2*
GO:0050817	coagulation	0.033	13	3	0.0005	0.16	*P2RY12, F5, F2RL2*
GO:0050878	reg body fluids	0.033	13	3	0.0005	0.16	*P2RY12, F5, F2RL2*
GO:0009611	wound response	0.033	14	4	0.001	0.36	*P2RY12, F5, F2RL2, PTAFR*
GO:0007243	protein kinase cascade	0.006	5	2	0.003	0.6	*P2RY12, SH2D3A*

All ontologies with a Fisher's exact p<0.01 are shown.

(1) The association p-value cut-off that gave the strongest enrichment.

(2) Number of genes with p-values below the p-value cut-off.

(3) Number of genes with the ontology.

To ascertain whether a single constituent coagulation phenotype was driving the associations, we tested whether the significant ontological enrichment observed using the reverse genetics model persisted when the most frequently-occurring diagnoses were omitted from the phenotype. When anti-coagulation use, strokes or venous thrombosis were excluded, no significantly-enriched ontologies were identified. In contrast, when myocardial infarction (MI) was excluded, the same pattern of ontological enrichment was observed but with slightly stronger enrichment p-values.

In sum, these analyses show that the mechanistic phenotype of vessel-occlusive disease was associated with genes related to blood coagulation.

### Mechanisms of tumorigenesis in whites

We next tested this approach with tumorigenesis, a highly mechanistically heterogeneous disease process [24]. We employed a larger population of 3,009 men and women of European ancestry (Table 1) and used 833 nsSNPs (748 genes). We first tested a single mechanistic phenotype comprised of all cancer diagnosis and treatment codes (see Tables S19, S20, S21, S22 in File S1 for associations between all nsSNPs and all cancer [ALL] mechanistic phenotype). However, no significantly enriched functionalities were identified (Tables S23, S22, S23, S24, S25, S26 in File S1). We hypothesized that this could be due to the extensive mechanistic heterogeneity underlying cellular transformation [24]. Consequently, we defined four mechanistic phenotypes representing different groupings of cancers in an effort to define aggregate phenotypes with sufficient cellular and mechanistic homogeneity to identify meaningful biological SNP associations (see Methods). In order to identify mechanisms common to all tumors, after computing association statistics for each of the four mechanistic phenotypes, p-values were pooled and analyzed for enrichment.

**Figure 3:**
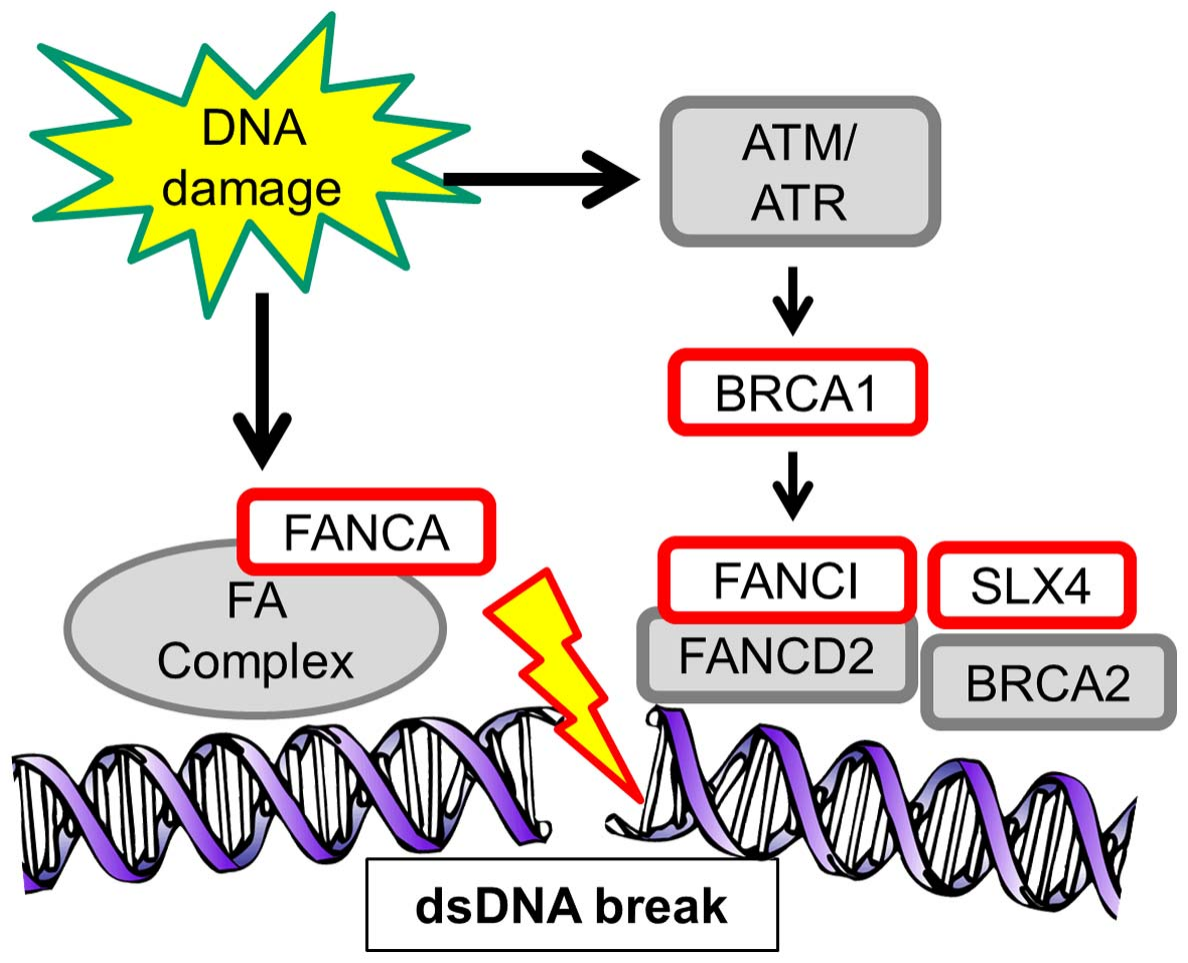
Double-stranded DNA repair pathway. Genes identified in the analyses are shown in red. When DNA is damaged, damage sensors promote recruitment and assembly of a repair complex comprised of Fanconi Anemia (FA) genes, BRCA1 and other proteins to the site of damage.

Both reverse genetics models were significantly enriched in functionalities related to DNA damage response and repair (p = 2×10^−5^ in both models) (Table 3 and Tables S27 and 28 in File S1). The enrichment was driven by the (*BRCA1, CHD1L* [25], *FANCA, POLG/FANCI, SLX4/FANCP* [26], *XRCC1* [27]) [28], [29] genes, a number of which participate in double-stranded DNA repair (Figure 3). In contrast, no significantly enriched ontologies were associated with the recessive model (Table S29 in File S1). The additive model did not show enrichment in DNA repair ontologies, but was significantly enriched in ontologies related to chromosome segregation during mitosis (p = 3.6×10^−6^) (Table 3 and Table S30 in File S1), driven by the genes *KIF25* [30], *PINX1* [31] and *FANCA*. A full GWAS using an additive model did not show any associations with genome-wide significant findings for any of the cancer mechanistic phenotypes (Figure S3). When we tested for enriched ontologies among the top 50 genes containing missense nsSNPs from the GWAS of the all cancer [ALL] phenotype, the top-ranked ontologies were related to DNA damage sensing and repair, but they were not statistically significant after adjusting for multiple testing (see Tables S31 and S32 in File S1 for the genes examined and enrichment analyses). The genes driving the top ontologies (*BRCA2* and *ATM*), while both involved in double-stranded DNA repair, were not the same as those associated with the reverse genetics models.

**Table 3:** Enriched ontologies for genes associated with the cancer phenotypes in whites.

Model/GO code	GO Term	P-value thres hold^1^	Genes below thres hold^2^	Genes with ontology^3^	Fisher's exact p-value	FDR p-value	Genes
**Reverse Genetics Model (>2 affected subjects)**							
GO:0006281	DNA repair	0.0098	27	60	0.00002	0.03	*FANCA, FANCI, BRCA1, SLX4, CHD1L, XRCC1*
GO:0033554	stress response	0.0098	27	7	0.00003	0.03	*FANCA, FANCI, PPP1R15B, BRCA1, SLX4, CHD1L, XRCC1*
GO:0006974	DNA damage response	0.0098	27	6	0.00003	0.03	*FANCA, FANCI, BRCA1, SLX4, CHD1L, XRCC1*
**Reverse Genetics Model 2 (>2 affected subjects and Fisher p<0.1)**							
GO:0006281	DNA repair	0.0098	27	6	0.00002	0.045	*FANCA, FANCI, BRCA1, SLX4, CHD1L, XRCC1*
GO:0006974	DNA damage response	0.0098	27	6	0.00003	0.045	*FANCA, FANCI, BRCA1, SLX4, CHD1L, XRCC1*
**Additive Model**							
GO:0000070	mitotic sister chromatid segregation	0.0003	2	2	0.000004	0.004	*KIF25, PINX1*
GO:0007059	chromosome segregation	0.0003	2	2	0.00004	0.03	*KIF25, PINX1*
GO:0000279	M phase	0.001	4	3	0.00005	0.03	*KIF25, PINX1, FANCA*
GO:0022403	cell cycle phase	0.001	4	3	0.00008	0.03	*KIF25, PINX1, FANCA*
GO:0000087	M phase of mitotic cell cycle	0.0003	2	2	0.00013	0.04	*KIF25, PINX1*
GO:0000280	nuclear division	0.0003	2	2	0.00013	0.04	*KIF25, PINX1*
GO:0007067	mitosis	0.0003	2	2	0.00013	0.04	*KIF25, PINX1*
GO:0048285	organelle fission	0.0003	2	2	0.00016	0.04	*KIF25, PINX1*
GO:0022402	cell cycle process	0.0010	4	3	0.00017	0.04	*KIF25, PINX1, FANCA*

All ontologies with an FDR p<0.05 are shown.

(1) The association p-value cut-off that gave the strongest enrichment.

(2) Number of genes with p-values below the p-value cut-off.

(3) Number of genes with the ontology.

We performed sensitivity analyses using the reverse genetics models by sequentially excluding one of the mechanistic phenotypes and performing the enrichment analysis. Only when the solid tumor (CA) phenotype was excluded did the significant enrichment in DNA repair ontologies persist. No ontologies were enriched when the HEM, MET and SKN phenotypes were excluded.

The association results between the nsSNPs in DNA repair genes and each cancer mechanistic phenotype for the 4 association models tested are summarized in Table 4. The significant associations from the additive model only matched the recessive and reverse genetics model for the FANCA and FANCI/POLG nsSNPs. In order to ascertain whether the DNA repair genes driving enrichment would demonstrate reproducible associations, we tested these nsSNPs using EMR-derived data from two independent data sets. The demographic characteristics for the replicate populations are shown in Table S33 in File S1. Sufficient subjects (> = 10 minor allele homozygotes) were available for the SNPs in five of the six genes. There were significant associations for *POLG*/*FANCI* (MET mechanistic phenotype, odds ratio [OR] = 2.4, p = 0.005) and for *CHD1L* (MET phenotype, OR = 4.1, p = 0.009) with a model comparing homozygotes and *FANCA* (SKN phenotype, OR = 1.7, p = 0.006) with an additive model (Table 5). Significant associations were only observed in the original mechanistic phenotype associated with the nsSNP, with the exception of *BRCA1* which was not associated with the HEM phenotype originally identified, but was associated with all cancers (OR = 8.1, p = 0.04), as 9 of the 10 homozygotes for this allele in the VESPA replication data set had a cancer diagnosis (Table 5).

**Table 4:** Association results for enriched DNA repair genes in whites.

					Recessive Model			Additive Model		
Phenotype		Reverse genetics P-value		Reverse genetics P-value	OR	95%CI	P	OR	95%CI	P
**BRCA1/rs1799950**										
ALL	0.13		0.091		2.6	(0.8–8.7)	0.12	1.1	(0.9–1.4)	0.31
CA	1		1		0.8	(0.3–2.8)	0.79	1.1	(0.9–1.4)	0.36
** HEM	0.009		0.008		4.9	(1.5–15.8)	0.008	1.1	(0.8–1.6)	0.38
MET	1		1		n/a	n/a	1.00	1.1	(0.8–1.5)	0.46
SKN	1		1		2.3	(0.3–18.4)	0.42	0.7	(0.4–1.4)	0.30
**CHD1L/rs2275249**										
ALL	0.31		0.17		0.9	(0.4–2.2)	0.85	1.1	(0.9–1.3)	0.50
CA	0.26		0.14		1.0	(0.4–2.4)	0.97	1.0	(0.8–1.2)	0.95
HEM	0.68		1		0.6	(0.1–2.7)	0.54	1.2	(0.9–1.6)	0.20
**MET	0.01		0.006		3.3	(1.3–8.2)	0.01	1.2	(0.9–1.6)	0.17
SKNSMB	1		1		1.3	(0.2–10.1)	0.78	1.0	(0.6–1.6)	0.98
**FANCA/rs17233497**										
ALL	0.19		0.036		1.6	(0.7–3.6)	0.22	1.1	(0.9–1.3)	0.19
CA	0.38		0.19		1.3	(0.6–3.0)	0.46	1.2	(1.0–1.4)	0.11
HEM	0.35		0.30		1.4	(0.5–4.1)	0.57	0.9	(0.7–1.2)	0.57
MET	0.22		0.11		1.4	(0.5–4.2)	0.54	1.0	(0.8–1.4)	0.85
**SKN	8.00E-04		8.00E-04		6.7	(2.5–18.3)	1.8E-04	1.9	(1.3–2.7)	0.001
**FANCI/POLG/rs3087374**										
ALL	0.20		0.06		1.1	(0.5–2.4)	0.90	1.0	(0.9–1.3)	0.65
CA	0.05		0.03		1.4	(0.6–3.2)	0.42	1.1	(0.9–1.3)	0.50
HEM	0.36		0.21		1.4	(0.5–4.3)	0.52	1.0	(0.7–1.3)	0.75
**MET	0.003		0.002		4.0	(1.6–9.6)	0.002	1.4	(1.0–1.8)	0.02
SKNSMB	1		1		1.3	(0.2–10.0)	0.78	1.1	(0.7–1.8)	0.70
**SLX4/rs3810813**										
ALL	0.048		0.03		3.3	(1.0–10.9)	0.047	1.1	(0.9–1.4)	0.42
**CA	0.01		0.005		4.4	(1.3–14.4)	0.01	1.1	(0.9–1.4)	0.43
HEM	1		1		n/a	n/a	1.00	1.0	(0.7–1.4)	0.86
MET	1		1		1.5	(0.3–7.1)	0.60	1.1	(0.8–1.5)	0.64
SKNSMB	1		1		n/a	n/a	1.00	0.8	(0.4–1.5)	0.41
**XRCC1/rs1799782**										
ALL	0.36		0.20		2.8	(1.0–8.3)	0.06	1.0	(0.8–1.3)	0.75
CA	0.27		0.16		2.1	(0.8–5.9)	0.15	1.0	(0.8–1.3)	0.82
HEM	0.35		0.30		1.8	(0.5–6.5)	0.38	1.0	(0.7–1.4)	0.97
MET	0.33		1		1.9	(0.5–6.9)	0.33	1.0	(0.7–1.4)	0.87
**SKN	0.01		0.01		7.3	(2.0–26.5)	0.002	1.2	(0.7–2.0)	0.56

Denotes the mechanistic phenotype driving the DNA repair enrichment.

‘n/a’ indicates there were no affected minor allele homozygotes for the phenotype. The value

**Table 5:** Association of DNA repair genes in independent data sets.

	Logistic regression comparing HZs^1^			Additive Model		
Phenotype	OR	95% CI	p-value	OR	95% CI	P
**eMERGE**						
** FANCA/rs17233497^2^**						
ALL	1.7	(0.7–4.0)	0.30	1.2	(0.9–1.4)	0.15
CA	1.5	(0.6–3.7)	0.42	1.1	(0.9–1.4)	0.32
HEM	2.0	(0.1–15.2)	0.84	1.2	(0.7–2.3)	0.51
MET	1.1	(0.02–7.1)	1.00	0.8	(0.5–1.5)	0.58
**SKN	4.1	(0.9–13.3)	0.06	1.7	(1.2–2.5)	0.006
** FANCI/POLG/rs3087374**						
ALL	1.4	(0.92–2.0)	0.13	1.0	(0.9–1.1)	0.82
CA	1.4	(0.9–2.1)	0.10	1.0	(0.9–1.1)	0.88
HEM	1.4	(0.6–3.5)	0.41	1.0	(0.8–1.2)	0.84
**MET	2.4	(1.3–4.4)	0.01	1.2	(1.0–1.4)	0.06
SKN	1.2	(0.4–3.8)	0.79	1.1	(0.9–1.4)	0.42
**VESPA**						
** BRCA1/rs1799950**						
ALL	8.1	(1.1–360.6)	0.04	1.0	(0.8–1.2)	0.96
CA	4.5	(0.9–44.4)	0.08	0.9	(0.8–1.1)	0.45
**HEM	0.7	(0.1–5.5)	1.00	1.1	(0.8–1.5)	0.42
MET	1.2	(0.1–6.5)	1.00	1.0	(0.8–1.3)	0.85
SKN	1.3	(0.2–6.1)	0.94	1.3	(0.9–2.0)	0.16
** CHD1L/rs2275249**						
ALL	2.3	(0.9–6.4)	0.09	0.9	(0.8–1.2)	0.62
CA	2.0	(0.8–5.0)	0.17	0.9	(0.7–1.2)	0.49
HEM	0.8	(0.2–2.9)	1.00	0.7	(0.5–1.0)	0.05
**MET	4.1	(1.4–10.8)	0.01	1.2	(0.9–1.6)	0.20
SKN	1.7	(0.6–4.5)	0.37	1.5	(1.0–2.2)	0.06
** FANCA/rs17233497**						
ALL	1.3	(0.6–2.9)	0.59	1.0	(0.8–1.1)	0.74
CA	1.3	(0.6–2.8)	0.67	1.0	(0.8–1.2)	0.94
HEM	1.7	(0.5–4.3)	0.40	1.0	(0.8–1.3)	0.85
MET	0.5	(0.1–1.7)	0.36	0.9	(0.7–1.1)	0.43
**SKN	1.4	(0.2–5.8)	0.92	0.8	(0.6–1.2)	0.35
** FANCI/POLG/rs3087374**						
ALL	0.9	(0.4–2.1)	0.90	0.9	(0.8–1.0)	0.15
CA	0.6	(0.2–1.5)	0.35	0.9	(0.8–1.1)	0.35
HEM	3.5	(1.3–8.7)	0.01	0.9	(0.7–1.2)	0.42
**MET	0.8	(0.2–2.4)	0.89	1.0	(0.8–1.3)	0.83
SKN	n/a	n/a	n/a	0.8	(0.5–1.2)	0.21
** SLX4/rs3810813**						
ALL	2.6	(0.5–16.1)	0.27	1.0	(0.8–1.2)	0.88
**CA	1.3	(0.3–5.9)	0.90	0.9	(0.8–1.1)	0.44
HEM	0.9	(0.02–7.5)	1.00	1.0	(0.7–1.3)	0.99
MET	1.5	(0.2–8.2)	0.86	0.9	(0.7–1.2)	0.59
SKN	n/a	n/a	n/a	0.6	(0.3–1.0)	0.07

(1) This model compared minor allele homozygotes to matched common allele homozygotes.

= 3,092 subjects). (2) Genotype data for this nsSNP was only available for one eMERGE site (n

## Discussion

In the present study, we evaluated the validity and utility of “mechanistic phenotypes” to identify cellular mechanisms underlying pathological thrombosis and tumorigenesis. We defined aggregate phenotypes comprised of EMR-derived diagnoses and treatments with potentially common underlying pathophysiological mechanisms. These phenotypes were utilized in conjunction with standard forward genetics statistical models and two reverse genetics models. The strength of the associations between the nsSNPs we evaluated and the mechanistic phenotypes were not stronger than would be expected by chance. Hence, we could not identify candidate nsSNPs on the basis of statistical outliers. To ascertain the biological relevance of the mechanistic phenotypes, we assessed whether the top-ranked nsSNPs had common functions consistent with the underlying biology of the phenotypes. We found that heritable variation in genes could elucidate with high specificity the most biologically-plausible physiological and cellular mechanisms underlying the diseases comprising our mechanistic phenotypes. Specifically, the thrombosis phenotype was only associated with hemostatic ontologies in AAs while the cancer phenotype was exclusively associated with chromosomal mechanisms related to DNA repair and chromosome segregation in whites. We found significant cancer associations for four of five DNA repair genes evaluated in independent data sets.

We hypothesized that mechanistic phenotypes could identify cellular mechanisms associated with a phenotype by identifying genes with functional commonalities. Support for this idea was recently demonstrated with a panel of 5 psychiatric diseases [32]. We first explored this approach using a heterogeneous collection of diseases arising due to occlusion of blood vessels. We found that the most enriched functionalities associated with this phenotype where consistent with a biologically-plausible disease mechanism, namely variation in platelet and coagulation factors. A sensitivity analysis of this phenotype showed that excluding myocardial infarction from the phenotype definition modestly improved the performance of this phenotype, suggesting that this disease may not be driven by the same mechanisms as the other constituent diseases.

In contrast to the thrombosis phenotype, when we grouped all cancers into a single phenotype, we did not observe significant enrichment in gene functionalities. Only when we divided cancers into distinct subgroups and then performed a pooled analysis of these subgroups did we find common functionalities. A major and largely exclusive pathologic mechanism underlying tumorigenesis is genomic instability [33] which facilitates the acquisition of the mutations required for uncontrolled growth and metastasis. Consistent with this known biology, our data suggest that genetic variation predisposing to genomic instability secondary to aberrant DNA repair and chromosomal segregation is associated with tumorigenesis. While a number of the genes associated with DNA repair were constituents of the common Fanconi Anemia/double-stranded DNA repair pathway [34], the individual genes were associated with distinct groupings of tumors, even in replication analyses. This would suggest that these genes exhibit tumor-type specificity and that a global cancer mechanistic phenotype may be too broad. *BRCA1*, however, stands in contrast to this notion, as it associated with both solid tumors and hematological tumors. Hence, further studies of a broad cancer mechanistic phenotype are needed to better clarify this issue.

Clinical disease taxonomies typically partition disease on the basis of anatomy, symptoms and clinico-pathological findings, rather than disease mechanisms [35]. GWAS studies that are based on these taxonomies may fail to identify significant biological mechanisms underlying a broad class of diseases because the effect size between a SNP and a single disease may be small. For instance, a SNP variant in a gene may be 100% penetrant for tumorigenesis (a disease mechanism), but may give rise to any of 30 different type of cancers, making the SNP appear poorly penetrant with respect to a given cancer. In this scenario, the relative risk between this SNP and a given cancer would be expected to be low, making an association difficult to identify by single-phenotype GWAS, even with large sample sizes. A mechanistic phenotype, as utilized in these analyses, represents an effort to map a clinical disease taxonomy to a mechanistic taxonomy by grouping diseases with common features. Hence, it represents a biologically-motivated approach to address the challenge of addressing type II pleiotropy, whereby a single molecular mechanism gives rise to multiple morphological expressions [36]. An optimal mechanistic phenotype, defined to have high sensitivity and specificity for a disease mechanism, would be expected to have increased power to detect the effect of a SNP variant, as compared to an individual constituent phenotype. It would also be expected to be minimally affected by the apparent variable penetrance of a given constituent phenotype. As the sensitivity analyses for our phenotypes have demonstrated, our mechanistic phenotypes are likely not optimally defined and will need continued refinement. However, these phenotypes are able to elucidate relevant biology, providing proof-of-concept for aggregative phenotyping approaches.

We explored two reverse genetics approaches to identify SNP-phenotype associations. For both the thrombosis analyses in AAs and cancer analyses in whites, the reverse genetics models were able to identify genes with common functionalities with higher specificity than a recessive genetic model. Based on the simulation analyses, these models are expected to show better enrichment when a SNP causes multiple cases of several constituent phenotypes or when a SNP causes multiple cases of a single phenotype. For the thrombosis mechanistic phenotype, this better performance, in conjunction with the fact that excluding any of the most frequent constituent diagnoses (except myocardial infarction) caused the models to fail to identify enriched ontologies, would suggest that the nsSNPs are causing multiple cases of the constituent diagnoses. One reason for the better performance of these models in the cancer data set is that the cancer groupings were chosen empirically based on common etiologies and environmental exposures, but may not represent optimal pathophysiological groupings. By focusing association testing on tumors over-represented among the minor allele homozygotes, the reverse genetics models may overcome some phenotype misspecification which can improve performance. The first reverse genetics model considered only those cancers that occurred in at least two homozygotes for the minor allele. The rationale for this restriction is that many known cancer-associated genes, even those involved in very basic cellular mechanisms such as *BRCA2*, demonstrate cancer-type specificity [28]. This model may be most useful when considering low frequency nsSNPs with relatively few minor allele homozygotes (as was the case with this study) as nsSNPs with large numbers of minor allele homozygotes are apt to have most tumors represented at least twice by chance. We also used a model that analyzed only those constituent cancers associated with a nsSNP with a Fisher's p<0.1. This model effectively takes a pheWAS-style analysis and determines whether there is an excess of mechanistically related diseases with a p-value below a pre-specified threshold. The relatively high p-value threshold was intentionally selected to remove diagnoses that were very likely present at levels expected by chance, but still included diagnoses that had sub-significant measures of association. Multiple sub-significant associations would be expected for highly pleiotropic nsSNPs. If this Fisher's p-value threshold were set to 1×10^−8^, this model would be equivalent to a standard PheWAS model that identifies individual SNP-phenotype associations that reach genome-wide significance. In sum, the advantage of a reverse genetics approach with mechanistic phenotypes is that it can facilitate identifying genotype and phenotype associations even if the mechanistic phenotype may not be defined with optimal specificity for a given SNP. In this context, the mechanistic phenotype restricts the choice of phenotypes considered in a reverse genetics analysis to those that may have a common cellular basis.

There are several limitations to this study. We utilized a convenience sample of subjects and relied on EMR data that were not accrued for research purposes. Hence, the extent and quality of phenotype ascertainment is variable. This would tend to cause non-differential misclassification of phenotypes which would be expected to attenuate any real associations. This limitation was likely more marked in our replication cancer cohorts since phenotypes were extracted solely from billing code data which represent an even more limited set of phenotypic data. Despite these limitations, we were still able to replicate associations in cancer analyses in whites. A limitation of these analyses was that there was no attempt to replicate the associations for genes enriched in ontologies related to blood coagulation. Our mechanism-discovery approach was also limited by the representation of ontologies in publicly available databases [37]. Hence, while other genes were identified that could suggest additional mechanisms of tumorigenesis [24], [38] such as evasion of immune surveillance (*TAP2* [39]), our approach may not have highlighted these processes because they were either poorly represented in the ontology databases or they did not pass the multiple-testing statistical thresholds. A challenge of a reverse genetics approach when used in conjunction with mechanistic phenotypes is that permutation testing is required to generate the null hypothesis and p-values, which can be computationally intensive. This is required because the phenotype represents an “optimized” set of diagnoses that was determined based on the observed diagnoses present among the minor allele homozygotes. In order to avoid estimating inappropriately low p-values (due to a “Winner's Curse” selection bias), the control group has to undergo the same optimization process. Hence, permutation testing is needed to estimate the null distribution associated with the optimization process.

In summary, we describe a mechanism-oriented phenotyping approach employing reverse genetics to identifying gene-phenotype associations using low frequency nsSNP variants. We applied this approach to two disease processes, thrombosis in AAs and tumorigenesis in whites, and identified cellular mechanisms and candidate genes associated with each process. This study demonstrates that these approaches can be used to leverage EMR data to assign functionality to genes and SNPs and provide new insights into genetic associations and disease risk.

## Supporting Information

Figure S1 **A mechanism-oriented phenotypic model.** Variations in a gene may disrupt physiological or cellular mechanisms, causing a myriad of clinical phenotypes. For example, a mutation in BRCA2 may manifest as breast cancer, ovarian cancer or prostate cancer; or in F5 as deep vein thrombosis, spontaneous abortions or pulmonary emboli. A mechanistic phenotype represents the collection of all potential clinical phenotypes that arise due to disruption of the cellular mechanism.
(PDF)

Figure S2 **QQ plot for an additive model between the thrombosis mechanistic phenotype and all overlapping SNPs on merged 1M-Duo and Omni1_QUAD genotyping platforms in African Americans.** The analysis includes all SNPs with a MAF>0.01 and HWE p-value>0.001.
(PDF)

Figure S3 **QQ plots for an additive model between the cancer mechanistic phenotypes and all SNPs on the Omni1_QUAD genotyping platforms in Whites.** Panel (a) all cancers [ALL]; panel (b) solid tumors [CA]; panel (c) hematological tumors [HEM]; panel (d) Metastatic tumors [MET]; and panel (e) skin cancers [SKN]. Plots include all SNPs with a MAF>0.01 and HWE p-value>0.001.
(PDF)

Figure S4 **ROC analyses for simulation studies for a reverse genetics model (>2 affected and Fisher**'**s p<0.1).** Analyses are based on 10,000 random samples of 13 subjects drawn from the thrombosis data set. ROC curves show sensitivity and specificities based on association p-values when one to five subjects were assigned to be affected with a constituent disease, as compared to association p-value with no additional subjects. Panel (a) is from simulations where subjects assigned a random disease and panel (b) is from simulations where subjects are assigned a disease already present among subjects in the random sample.
(PDF)

Figure S5 **ROC analyses for simulation studies.** Panels (a-c) are for simulations where additional affected subjects were assigned a disease with likelihood proportional to the prevalence of the disease among the thrombosis constituent phenotypes and panels (d–f) are for simulations where all five additional subjects are assigned to have the same disease, randomly selected. ROC curves are for a recessive model (panels (a) and (c)), a reverse genetics model (>2 affected) (panels (b) and (d)) and a reverse genetics (>2 affected and Fisher's p<0.1) (panels (c) and (e)).
(PDF)

File S1 **Table S1. Characteristics of the nsSNPs analyzed in the thrombosis study in AAs. Table S2. Characteristics of the nsSNPs analyzed in the cancer study in whites. Table S3. SNP selection and exclusion statistics. Table S4. Constituent phenotype ICD-9 grouping for the thrombosis mechanistic phenotype.** Each row contains a diagnosis cluster and the ICD-9 codes mapped to that cluster. These groupings are similar to the Clinical Classification System for diagnosis codes, but some of the problem headings in the original classification system were subdivided into smaller groups of related ICD-9 codes. **Table S5. ICD-9 and cancer mechanistic phenotype groupings.** Each row contains a diagnosis cluster and the ICD-9 codes mapped to that cluster. These groupings are similar to the Clinical Classification System for diagnosis codes, but some of the problem headings in the original classification system were subdivided into smaller groups of related ICD-9 codes. Also shown are the mechanistic phenotypes (meta-groupings) that the diagnosis cluster was assigned to. **Table S6. List of all functional ontologies associated with genes in the thrombosis data set. Table S7. List of all functional ontologies associated with genes in the cancer data set. Table S8. Type I error rates from simulations using the thrombosis data set.** Type I error rates for the thrombosis phenotype are based on 1,000 randomizations of the 433 SNPs in the thrombosis data set. Type I error rates for the cancer data set are based on 500 randomizations of 833 SNPs in the cancer data set. For both reverse genetics models, the type 1 error represents the proportion of with p-values below the indicated threshold. **Table S9. Association p-values between the thrombosis mechanistic phenotype and nsSNPs using a reverse genetics model (> = 2 affected subjects). Table S10. Association p-values between the thrombosis mechanistic phenotype and nsSNPs using a reverse genetics model (> = 2 affected subjects and Fisher p<0.1). Table S11. Results for ontological analyses of genes with nsSNPs associated with the thrombosis mechanistic phenotype using the reverse genetics model (> = 2 affected subjects).** The upper portion of the table shows are all functional ontologies associated with 2 or more genes. The “P-value threshold” column is the association p-value cut-off that gave the strongest enrichment. The “Genes below threshold” column is number of genes with p-values below this threshold. The “Genes with ontology” column is the number of genes that are assigned the ontology. The second table (below) shows the specific genes associated with each ontology. **Table S12. Results for ontological analyses of genes with nsSNPs associated with the thrombosis mechanistic phenotype using the reverse genetics model (> = 2 affected subjects and Fisher's p<0.1).** The upper portion of the table shows are all functional ontologies associated with 2 or more genes. The “P-value threshold” column is the association p-value cut-off that gave the strongest enrichment. The “Genes below threshold” column is number of genes with p-values below this threshold. The “Genes with ontology” column is the number of genes that are assigned the ontology. The second table (below) shows the specific genes associated with each ontology. **Table S13. Association p-values between the thrombosis mechanistic phenotype and nsSNPs using a recessive genetic model.** (OR = odds ratio; SE = standard error; L95 and U95 are the upper and lower bounds of the 95% confidence interval). **Table S14. Results for ontological analyses of genes with nsSNPs associated with the thrombosis mechanistic phenotype for the recessive model.** The upper portion of the table shows are all functional ontologies associated with 2 or more genes. Only those genes containing a nsSNP with an OR>1 were used in enrichment analyses. The “P-value threshold” column is the association p-value cut-off that gave the strongest enrichment. The “Genes below threshold” column is number of genes with p-values below this threshold. The “Genes with ontology” column is the number of genes that are assigned the ontology. The second table (below) shows the specific genes associated with each ontology. **Table S15. Association p-values between the thrombosis mechanistic phenotype and nsSNPs using an additive genetic model.** (OR = odds ratio; SE = standard error; L95 and U95 are the upper and lower bounds of the 95% confidence interval). **Table S16. Results for ontological analyses of genes with nsSNPs associated with the thrombosis mechanistic phenotype for the additive model.** The upper portion of the table shows are all functional ontologies associated with 2 or more genes. Only those genes containing a nsSNP with an OR>1 were used in enrichment analyses. The “P-value threshold” column is the association p-value cut-off that gave the strongest enrichment. The “Genes below threshold” column is number of genes with p-values below this threshold. The “Genes with ontology” column is the number of genes that are assigned the ontology. The second table (below) shows the specific genes associated with each ontology. **Table S17. Top ranked nsSNPs from a GWAS of the thrombosis mechanistic phenotype in AAs.** The GWAS was conducted using an additive model and adjusted for gender, age and genotyping platform. The GWAS was conducted on the intersection of nsSNPs on the Illumina 1M-Duo and Omni1_QUAD platforms. There were 8,913 nsSNPs (n = 5,391 genes) with a MAF>0.01 and HWE>0.001. Shown are the nsSNP associations for the top 50 genes with the strongest associations. (OR = odds ratio; SE = standard error; L95 and U95 are the upper and lower bounds of the 95% confidence interval). **Table S18. Results for ontological analyses of genes using the top 50 genes containing nsSNPs associated with an additive model.** See supplemental table 17 for the list of genes tested. The upper portion of the table shows are all functional ontologies associated with 2 or more genes. Only those genes containing a nsSNP with an OR>1 were used in enrichment analyses. The “P-value threshold” column is the association p-value cut-off that gave the strongest enrichment. The “Genes below threshold” column is number of genes with p-values below this threshold. The “Genes with ontology” column is the number of genes that are assigned the ontology. The second table (below) shows the specific genes associated with each ontology. **Table S19. nsSNP association p-values between the cancer mechanistic phenotypes and nsSNPs using a reverse genetics model (> = 2 affected subjects).** Table S20. nsSNP association p-values between the cancer mechanistic phenotypes and nsSNPs using a reverse genetics model (> = 2 affected subjects and Fisher p<0.1). **Table S21. nsSNP association p-values between the cancer mechanistic phenotypes and nsSNPs using a recessive genetic model.** (OR = odds ratio; SE = standard error; L95 and U95 are the upper and lower bounds of the 95% confidence interval). **Table S22. nsSNP association p-values between the cancer mechanistic phenotypes and nsSNPs using an additive genetic model.** (OR = odds ratio; SE = standard error; L95 and U95 are the upper and lower bounds of the 95% confidence interval). **Table S23. Results for ontological analyses of genes with nsSNPs associated with the all cancers [ALL] cancer mechanistic phenotype using the reverse genetics model (> = 2 affected subjects).** The upper portion of the table shows are all functional ontologies associated with 2 or more genes. The “P-value threshold” column is the association p-value cut-off that gave the strongest enrichment. The “Genes below threshold” column is number of genes with p-values below this threshold. The “Genes with ontology” column is the number of genes that are assigned the ontology. The second table (below) shows the specific genes associated with each ontology. **Table S24. Results for ontological analyses of genes with nsSNPs associated with the all cancers [ALL] cancers mechanistic phenotype using the reverse genetics model (> = 2 affected subjects and Fisher's p<0.1).** The upper portion of the table shows are all functional ontologies associated with 2 or more genes. The “P-value threshold” column is the association p-value cut-off that gave the strongest enrichment. The “Genes below threshold” column is number of genes with p-values below this threshold. The “Genes with ontology” column is the number of genes that are assigned the ontology. The second table (below) shows the specific genes associated with each ontology. **Table S25. Results for ontological analyses of genes with nsSNPs associated with the with the all cancers [ALL] cancers mechanistic phenotype for the recessive model.** The upper portion of the table shows are all functional ontologies associated with 2 or more genes. Only those genes containing a nsSNP with an OR>1 were used in enrichment analyses. The “P-value threshold” column is the association p-value cut-off that gave the strongest enrichment. The “Genes below threshold” column is number of genes with p-values below this threshold. The “Genes with ontology” column is the number of genes that are assigned the ontology. The second table (below) shows the specific genes associated with each ontology. **Table S26. Results for ontological analyses of genes with nsSNPs associated with the with the all cancers [ALL] cancers mechanistic phenotype for the additive model.** The upper portion of the table shows are all functional ontologies associated with 2 or more genes. Only those genes containing a nsSNP with an OR>1 were used in enrichment analyses. The “P-value threshold” column is the association p-value cut-off that gave the strongest enrichment. The “Genes below threshold” column is number of genes with p-values below this threshold. The “Genes with ontology” column is the number of genes that are assigned the ontology. The second table (below) shows the specific genes associated with each ontology. **Table S27. Results for ontological analyses of genes with nsSNPs associated with the cancer mechanistic phenotypes (CA, HEM, MET or SKN) using the reverse genetics model (> = 2 affected subjects).** The upper portion of the table shows are all functional ontologies associated with 2 or more genes. The “P-value threshold” column is the association p-value cut-off that gave the strongest enrichment. The “Genes below threshold” column is number of genes with p-values below this threshold. The “Genes with ontology” column is the number of genes that are assigned the ontology. The second table (below) shows the specific genes associated with each ontology. **Table S28. Results for ontological analyses of genes with nsSNPs associated with the cancer mechanistic phenotypes (CA, HEM, MET or SKN) using the reverse genetics model (> = 2 affected subjects and Fisher's p<0.1).** The upper portion of the table shows are all functional ontologies associated with 2 or more genes. The “P-value threshold” column is the association p-value cut-off that gave the strongest enrichment. The “Genes below threshold” column is number of genes with p-values below this threshold. The “Genes with ontology” column is the number of genes that are assigned the ontology. The second table (below) shows the specific genes associated with each ontology. **Table S29. Results for ontological analyses of genes with nsSNPs associated with the with the cancer mechanistic phenotypes (CA, HEM, MET or SKN) for the recessive model.** The upper portion of the table shows are all functional ontologies associated with 2 or more genes. Only those genes containing a nsSNP with an OR>1 were used in enrichment analyses. The “P-value threshold” column is the association p-value cut-off that gave the strongest enrichment. The “Genes below threshold” column is number of genes with p-values below this threshold. The “Genes with ontology” column is the number of genes that are assigned the ontology. The second table (below) shows the specific genes associated with each ontology. **Table S30. Results for ontological analyses of genes with nsSNPs associated with the with the cancer mechanistic phenotypes (CA, HEM, MET or SKN) for the additive model.** The upper portion of the table shows are all functional ontologies associated with 2 or more genes. Only those genes containing a nsSNP with an OR>1 were used in enrichment analyses. The “P-value threshold” column is the association p-value cut-off that gave the strongest enrichment. The “Genes below threshold” column is number of genes with p-values below this threshold. The “Genes with ontology” column is the number of genes that are assigned the ontology. The second table (below) shows the specific genes associated with each ontology. **Table S31. Top ranked nsSNPs from a GWAS of the “all cancer**' **[ALL] mechanistic phenotype in whites.** The GWAS was conducted using an additive model and adjusted for gender and age. There were 16,378 nsSNPs (n = 7,896 genes) with a MAF>0.01 and HWE>0.001 evaluated. Shown are the nsSNP associations for the top 50 genes with the strongest associations. (OR = odds ratio; SE = standard error; L95 and U95 are the upper and lower bounds of the 95% confidence interval). **Table S32. Results for ontological analyses of genes using the top 50 genes containing nsSNPs associated with an additive model.** See Table S31 for the list of genes tested. The upper portion of the table shows are all functional ontologies associated with 2 or more genes. Only those genes containing a nsSNP with an OR>1 were used in enrichment analyses. The “P-value threshold” column is the association p-value cut-off that gave the strongest enrichment. The “Genes below threshold” column is number of genes with p-values below this threshold. The “Genes with ontology” column is the number of genes that are assigned the ontology. The second table (below) shows the specific genes associated with each ontology. **Table S33. Demographic characteristics of the subjects in the eMERGE and VESPA replication sets.**
(XLSX)
