# Supplementary figures and images for: Mechanistic Phenotypes: An Aggregative Phenotyping Strategy to Identify Disease Mechanisms Using GWAS Data
Source: PLoS One. 2013 Dec 12;8(12):e81503. doi: 10.1371/journal.pone.0081503 (PMC3861317; doi:10.1371/journal.pone.0081503)

# Supplemental figure 1

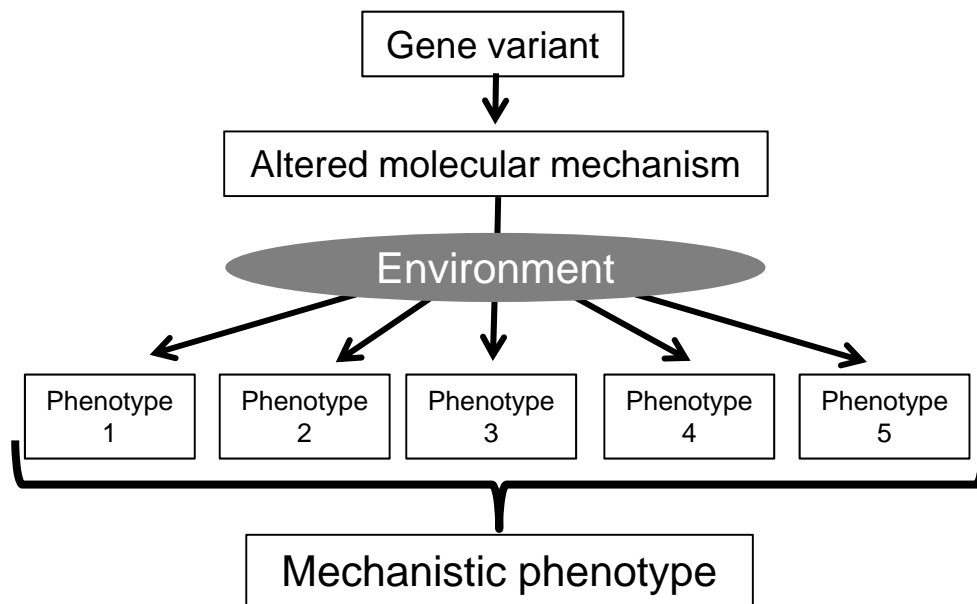

Supplement: Figure S1 — A mechanism-oriented phenotypic model. Variations in a gene may disrupt physiological or cellular mechanisms, causing a myriad of clinical phenotypes. For example, a mutation in BRCA2 may manifest as breast cancer, ovarian cancer or prostate cancer; or in F5 as deep vein thrombosis, spontaneous abortions or pulmonary emboli. A mechanistic phenotype represents the collection of all potential clinical phenotypes that arise due to disruption of the cellular mechanism. (PDF) [file pone.0081503.s001.pdf]

## Supplemental figure 2

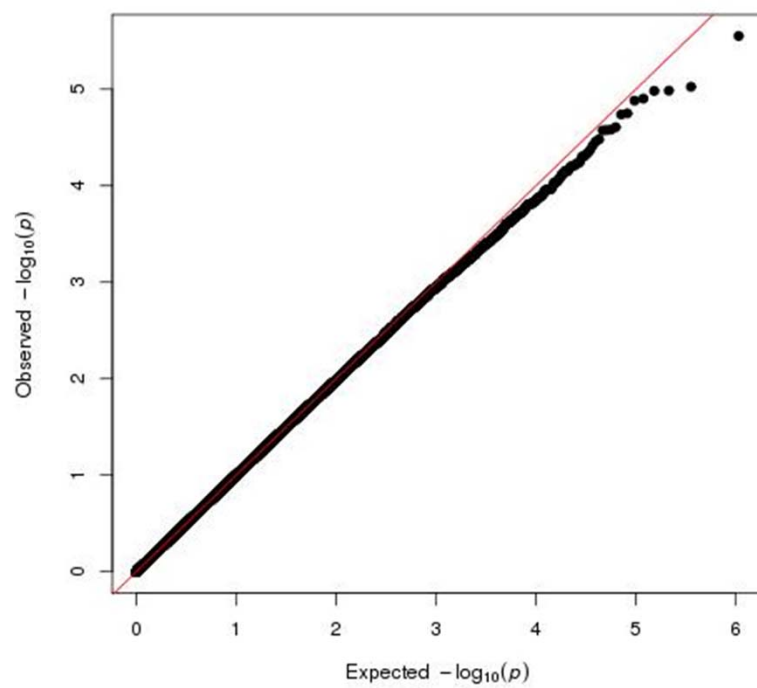

Supplement: Figure S2 — QQ plot for an additive model between the thrombosis mechanistic phenotype and all overlapping SNPs on merged 1M-Duo and Omni1_QUAD genotyping platforms in African Americans. The analysis includes all SNPs with a MAF>0.01 and HWE p-value>0.001. (PDF) [file pone.0081503.s002.pdf]

# Supplemental figure 3

a

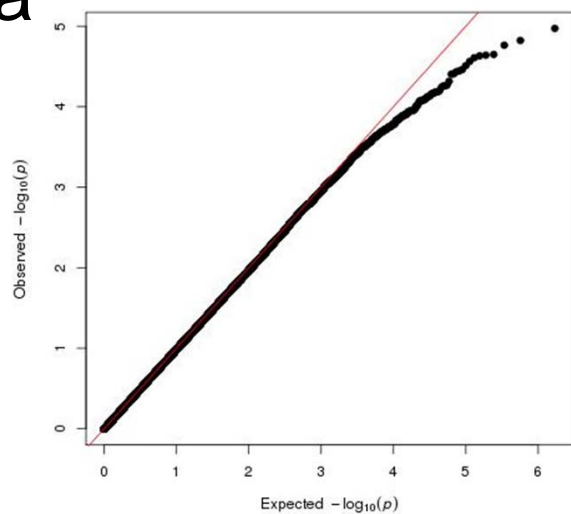

b

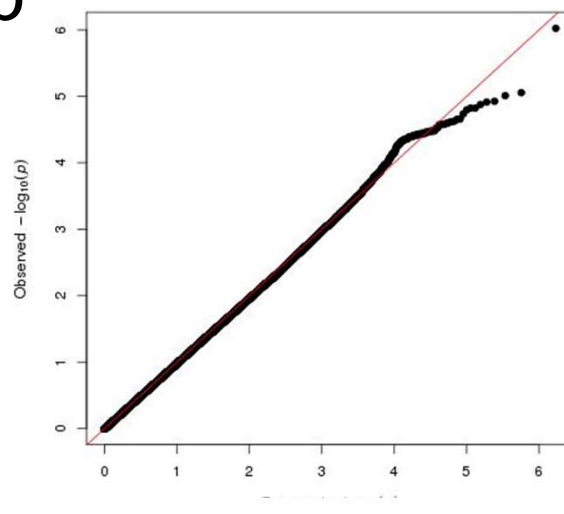

c

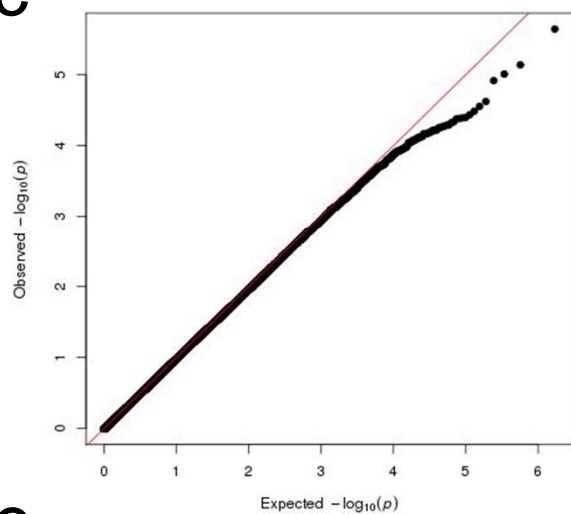

d

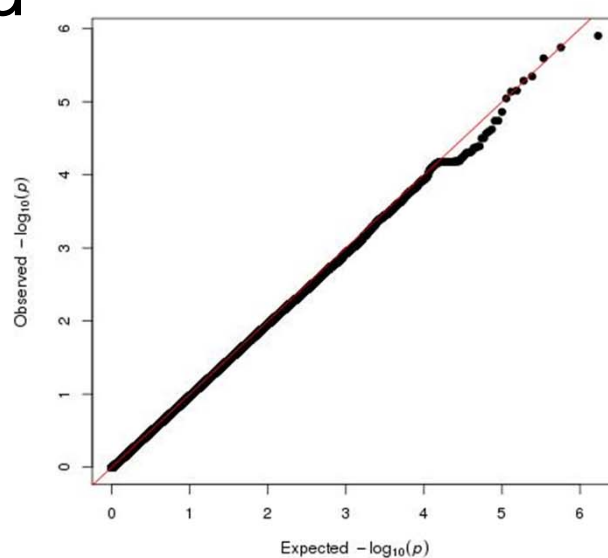

e

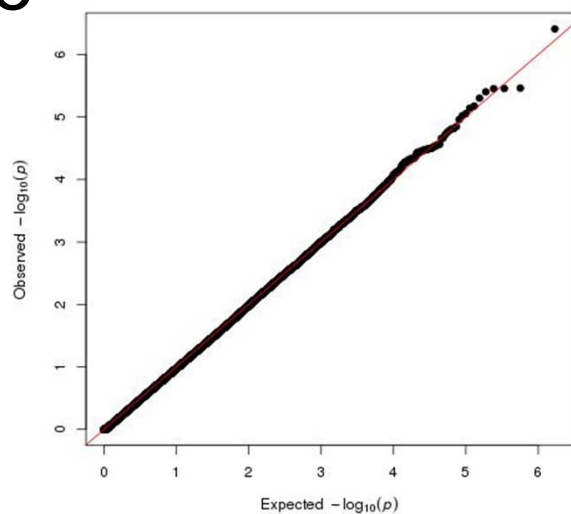

Supplement: Figure S3 — QQ plots for an additive model between the cancer mechanistic phenotypes and all SNPs on the Omni1_QUAD genotyping platforms in Whites. Panel (a) all cancers [ALL]; panel (b) solid tumors [CA]; panel (c) hematological tumors [HEM]; panel (d) Metastatic tumors [MET]; and panel (e) skin cancers [SKN]. Plots include all SNPs with a MAF>0.01 and HWE p-value>0.001. (PDF) [file pone.0081503.s003.pdf]

# Supplemental figure 4

a

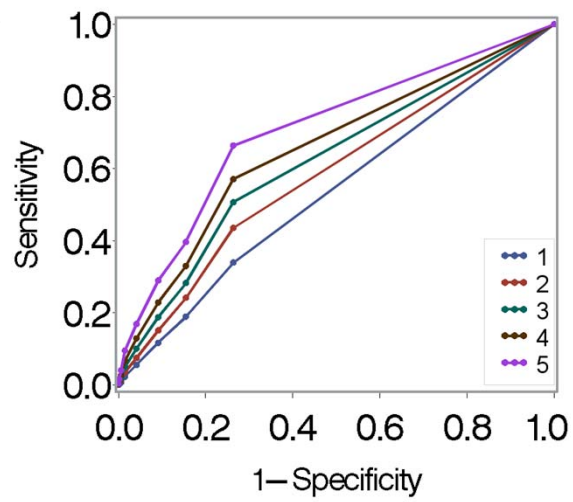

b

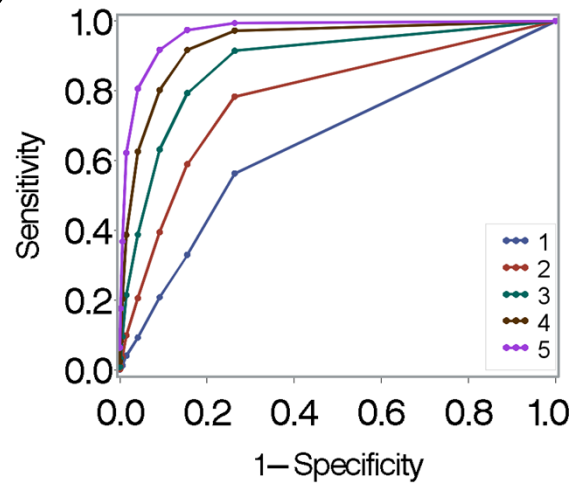

Supplement: Figure S4 — ROC analyses for simulation studies for a reverse genetics model (>2 affected and Fisher's p<0.1). Analyses are based on 10,000 random samples of 13 subjects drawn from the thrombosis data set. ROC curves show sensitivity and specificities based on association p-values when one to five subjects were assigned to be affected with a constituent disease, as compared to association p-value with no additional subjects. Panel (a) is from simulations where subjects assigned a random disease and panel (b) is from simulations where subjects are assigned a disease already present among subjects in the random sample. (PDF) [file pone.0081503.s004.pdf]

# Supplemental figure 5

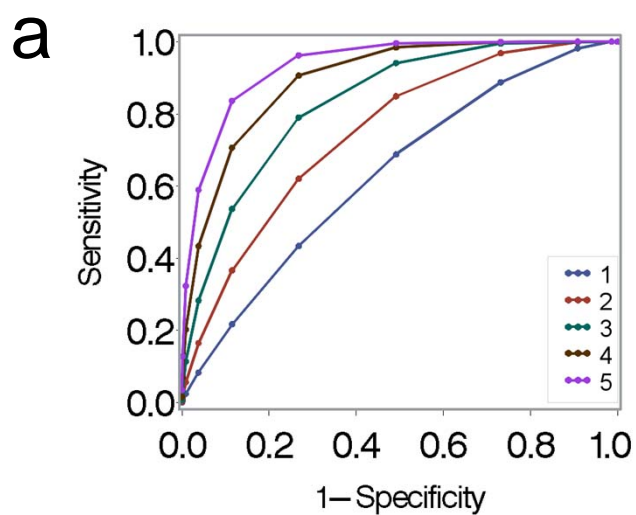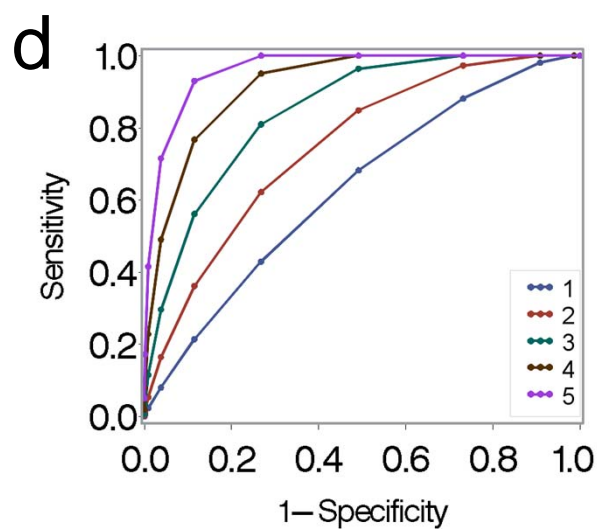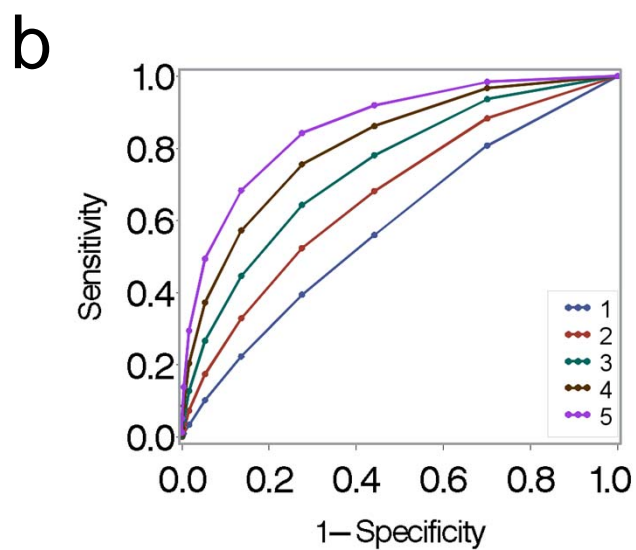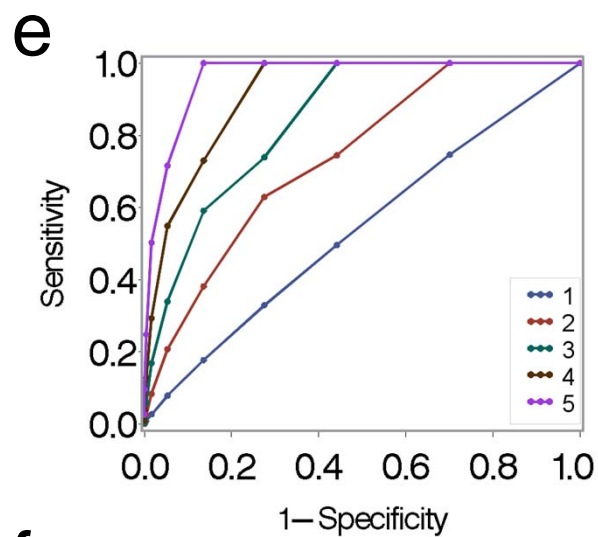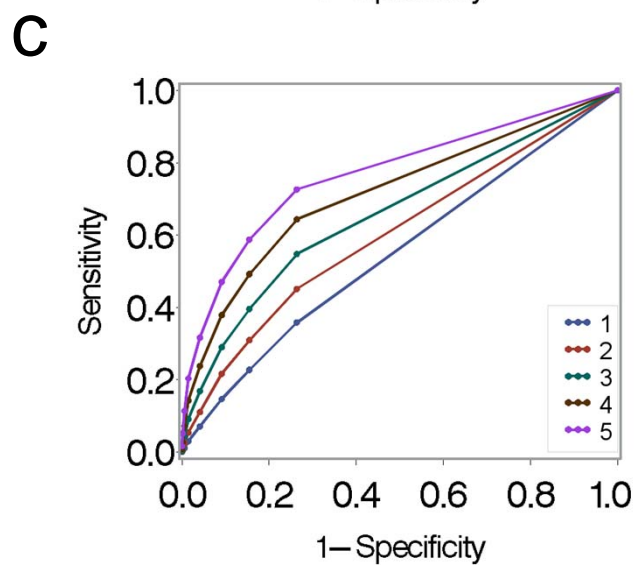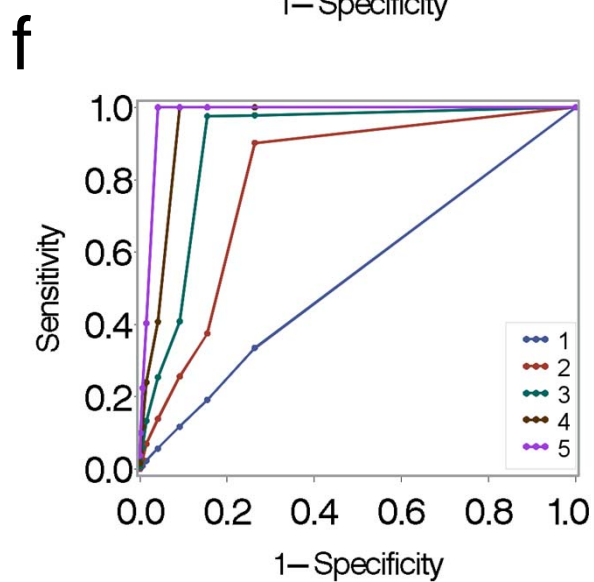

Supplement: Figure S5 — ROC analyses for simulation studies. Panels (a-c) are for simulations where additional affected subjects were assigned a disease with likelihood proportional to the prevalence of the disease among the thrombosis constituent phenotypes and panels (d–f) are for simulations where all five additional subjects are assigned to have the same disease, randomly selected. ROC curves are for a recessive model (panels (a) and (c)), a reverse genetics model (>2 affected) (panels (b) and (d)) and a reverse genetics (>2 affected and Fisher's p<0.1) (panels (c) and (e)). (PDF) [file pone.0081503.s005.pdf]
